# Substrate binding in the mitochondrial ADP/ATP carrier is a step-wise process guiding the structural changes in the transport cycle

**DOI:** 10.1038/s41467-022-31366-5

**Published:** 2022-06-23

**Authors:** Vasiliki Mavridou, Martin S. King, Sotiria Tavoulari, Jonathan J. Ruprecht, Shane M. Palmer, Edmund R. S. Kunji

**Affiliations:** Medical Research Council Mitochondrial Biology Unit, University of Cambridge, Cambridge Biomedical Campus, Keith Peters Building, Hills Road, Cambridge, CB2 0XY UK

**Keywords:** Bioenergetics, Structural biology, Biophysical methods, Permeation and transport, Mitochondria

## Abstract

Mitochondrial ADP/ATP carriers import ADP into the mitochondrial matrix and export ATP to the cytosol to fuel cellular processes. Structures of the inhibited cytoplasmic- and matrix-open states have confirmed an alternating access transport mechanism, but the molecular details of substrate binding remain unresolved. Here, we evaluate the role of the solvent-exposed residues of the translocation pathway in the process of substrate binding. We identify the main binding site, comprising three positively charged and a set of aliphatic and aromatic residues, which bind ADP and ATP in both states. Additionally, there are two pairs of asparagine/arginine residues on opposite sides of this site that are involved in substrate binding in a state-dependent manner. Thus, the substrates are directed through a series of binding poses, inducing the conformational changes of the carrier that lead to their translocation. The properties of this site explain the electrogenic and reversible nature of adenine nucleotide transport.

## Abstract (web summary)

The mitochondrial ADP/ATP carrier transports adenine nucleotides. Here, authors identify the residues involved in substrate binding. One set forms the central substrate binding site and two asparagine/arginine pairs help to guide the substrates during the transport cycle.

## Introduction

The viability and function of eukaryotic cells rely on the transport of metabolites and ions across the impermeable mitochondrial inner membrane in order to sustain the metabolic pathways of the mitochondrion and cytosol and to provide compounds for organellar and cellular maintenance. The majority of these compounds are transported by the mitochondrial carrier family (SLC25), which is the largest solute carrier family in humans^1–3^. The mitochondrial ADP/ATP carrier, also called adenine nucleotide translocase, carries out one of the most prolific transport steps of the human body. The carrier imports cytosolic ADP into the mitochondrial matrix for the conversion to ATP by the ATP synthase and it exports ATP to the intermembrane space, which is confluent with the cytoplasm, to power the energy-requiring processes of the cell, as an integral part of oxidative phosphorylation^4^. The carrier cycles between two states, which are conventionally called the matrix-open state and the cytoplasmic-open state, because of the orientation of the substrate-binding site towards these compartments^5,6^. Functional and structural studies have been aided by the availability of state-specific inhibitors. The carrier is locked in a cytoplasmic-open state by atractyloside (ATR)^7^ and carboxyatractyloside (CATR)^8,9^, whereas it is locked in a matrix-open state by bongkrekic acid (BKA)^10–12^. Both inhibitor-bound states are abortive because inhibition is achieved by preventing substrate binding and by trapping the carrier in a structural conformation that is not part of the transport cycle^5,6^.

Like most SLC25 members, the ADP/ATP carrier consists of three homologous sequence repeats of ~100 amino acids each^13^, which fold into a three-fold pseudo-symmetrical structure with a central translocation pathway for the substrates^14^. The atomic structure of the bovine ADP/ATP carrier in the CATR-bound cytoplasmic-open state showed that each sequence repeat encodes a domain consisting of an odd-numbered transmembrane α-helix (H1, H3, H5), a loop containing a short helix (h12, h34, h56) running parallel to the membrane on the matrix side and an even-numbered transmembrane α-helix (H2, H4, H6)^15^ (Supplementary Fig. 1). This basic topology was confirmed by the atomic structures of the fungal ADP/ATP carriers locked in the CATR-bound cytoplasmic-open state^16^ and in the BKA-bound matrix-open state^17^.

In each of the three domains, the odd-numbered helix, matrix helix and the N-terminal part of the even-numbered helix to the ‘contact point’^17–19^, make up the core element, whereas the C-terminal part of the even-numbered helix forms the gate element^17^ (Supplementary Fig. 1). Interconversion between the two states occurs in an alternating way^20^ and involves extensive movements of the six structural elements, making the ADP/ATP carrier the most dynamic solute transporter identified to date^5,17^. The three gate elements open and close the cytoplasmic side of the carrier, while the three core elements close and open the matrix side, alternately. The opening and closing are regulated by the disruption and formation of two salt bridge networks and braces: the matrix salt bridge network of the [PS]x[DE]xx[KR] motif^15,16^ and glutamine brace^16^ on the core elements and the cytoplasmic salt bridge network of the [FY][DE]xx[RK] motif^17,20,21^ and tyrosine braces^17^ on the gate elements (Supplementary Fig. 1). The coordinated movement of these six structural elements is driven by substrate binding and release as the only direct source of energy input^22,23^.

In the transport cycle, substrate binding and conformational changes are interdependent processes, meaning that there must be a continuous series of highly dynamic substrate-bound states: from the cytoplasmic-open state, via an occluded state, to the matrix-open state, and back. During this process, the ADP and ATP molecules may adopt many different conformers, as the carrier switches between different conformations. Therefore, it is necessary to untangle this complex process, before we can address it by direct structural analyses.

Biochemical studies that used non-transportable nucleotide analogues, conducted prior to the availability of structures, proposed substrate-binding sites in the matrix loops^24–27^ and C-terminal region^27^. Later, sequence analyses, which used chemical and distance constraints^18,19^ or deviation of pseudo-symmetry^20^ as concepts, pointed towards a location in the central part of the cavity, which included three ‘contact points’^18,19^ (Supplementary Fig. 1). In contrast, molecular dynamics simulations highlighted substrate interactions throughout the translocation pathway^28–31^. Evidently, the different studies have not pointed to a consensus location for the substrate-binding site, and thus its experimental determination will be an essential first step in addressing the key issues of substrate-binding and conformational coupling of the mitochondrial ADP/ATP carrier.

**Fig. 1:**
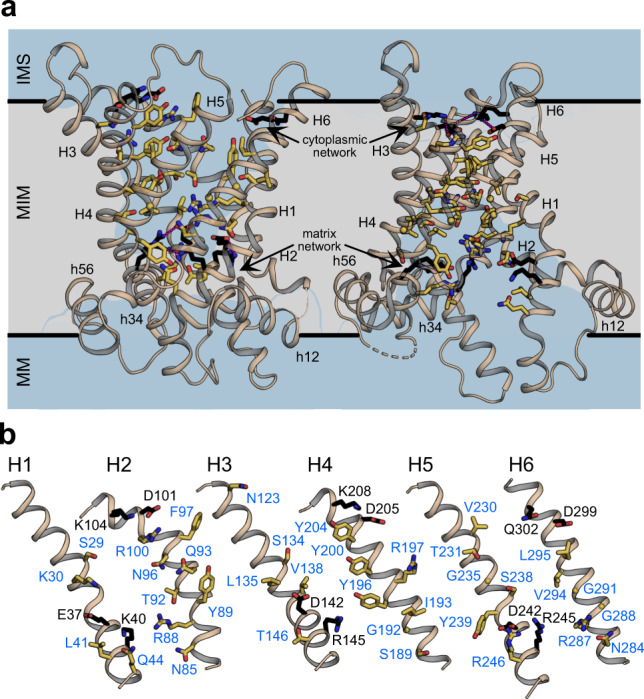
The amino acid residues of the translocation pathway analysed in the study. **a** Membrane view of homology model of TtAac in the cytoplasmic-open state (left) and the experimentally determined structure of TtAac in the matrix-open state (right) (PDB code: 6gci chain A). The residues analysed in this study are indicated as yellow sticks, whereas the residues of the matrix and cytoplasmic networks as black sticks, with ionic interactions shown as magenta dashes. The water-accessible surfaces, determined by HOLE^59^, are shown in transparent blue. **b** Residues of the transmembrane helices lining the translocation pathway, analysed in this study, are labelled in blue, and the residues of the salt bridge networks in black.

Here, we provide the direct experimental identification of the residues involved in substrate binding by combining functional assays with binding studies, using alanine replacement mutants of the substrate translocation pathway (Fig. 1). Our approach identifies all residues that interact with the substrates, irrespective of the conformational state. The identification of the binding site residues provides the framework to resolve important molecular details of the binding and transport mechanism. Importantly, the residues involved in ADP and ATP binding are the same, explaining the fully reversible electrogenic nature of transport.

## Results

### Most residues in the translocation pathway are critical for function

In order to identify residues that could be involved in substrate binding, we analysed the water-accessible residues in the mitochondrial ADP/ATP carrier from the fungus *Thermothelomyces thermophila* (TtAac), which is more stable in detergents than the yeast orthologues^32^. To identify all candidate residues, we used the experimentally determined matrix-open structure of TtAac^17^ and a comparative homology model of the cytoplasmic-open state, based on the resolved structures of the cytoplasmic-open state (PDB entries 1okc, 4c9h, 4c9q and 4c9j). In total, 36 residues were identified in the translocation pathway, encompassing all solvent-accessible residues with the exception of the two salt bridge networks, which are universally conserved, irrespective of the substrate specificity, and have a defined role in the mechanism^15–17,20,21^ (Fig. 1). Each of the selected residues was replaced by alanine, generating a set of 36 variants.

**Fig. 2:**
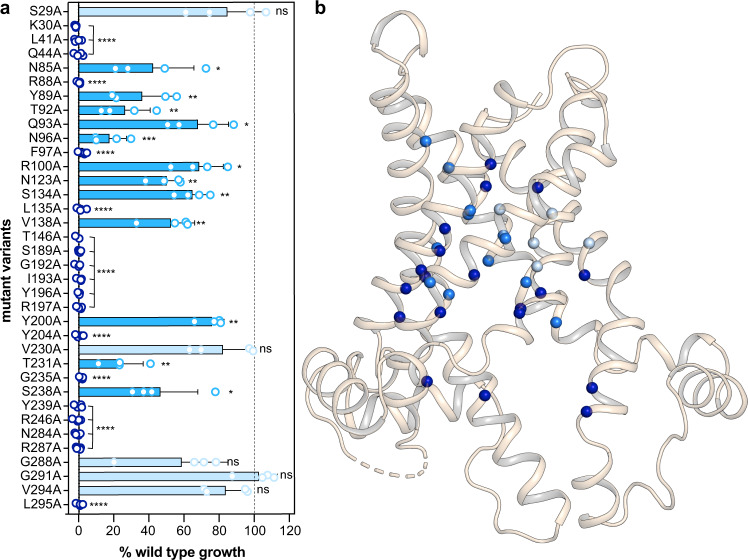
Most residues of the translocation pathway are critical for function. **a** Percentage of functional complementation of the WB-12 strain expressing TtAac variants compared to expressing the wild-type carrier (100% complementation), as determined by densitometry of serial dilution spot tests on YPG medium (Supplementary Fig. 2). The bars and error bars represent mean and standard deviation of four independent experiments. Significance analysis was performed with two-tailed one-sample *t*-tests, as described in “Methods” (*p* > 0.05, ns; *p* ≤ 0.05, *; *p* ≤ 0.01, **; *p* ≤ 0.001, ***; *p* ≤ 0.0001, ****). Colours represent the three observed growth properties: growth not affected significantly (ns), light blue; growth significantly affected (0.5 ≥ *p* > 0.0001), marine; no growth (*p* ≤ 0.0001), dark blue. **b** Position of the analysed residues in the matrix-open BKA-bound TtAac structure (PDB code: 6gci chain A), shown as a cartoon representation. Spheres represent the CA carbon atoms and are colour-coded as described in **a**. Source data for this figure are provided as a Source Data file.

First, we performed functional complementation studies using the transport-deficient WB-12 strain of *Saccharomyces cerevisiae*^33^. The expression of wild-type TtAac fully restored the growth of WB-12 (100% complementation), while the control, containing an empty vector, showed no growth (Supplementary Fig. 2). Of the 36 alanine variants, only five complemented growth to a similar extent as the wild type, while 19 showed no growth at all (<3%) and another 12 showed significantly reduced growth (ranging from 18 to 76%) (Fig. 2a, Supplementary Fig. 2). The 19 residues essential for function are dispersed throughout the central cavity (Fig. 2b) and are highly conserved among ADP/ATP carrier orthologues (Supplementary Fig. 3). The charged residues K30, R88, R197, R246 and R287 have been previously shown to be important for growth and transport activity in other orthologues^34–39^. These data showed that most residues in the translocation pathway are important for efficient ADP/ATP exchange.

### Detection of residues involved in carrier-ligand interactions

Complementation assays cannot determine which aspect of the structure or function is affected by the mutation. The low binding affinity of the substrates (μM range^23^), a crucial aspect of the transport mechanism, precludes the use of binding assays. As complementation assays cannot identify residues involved specifically in substrate binding, we used a thermostability shift assay, which monitors the unfolding of a protein population with the thiol-reactive probe 7‐diethylamino‐3‐(4‐maleimidophenyl)‐4‐methyl coumarin (CPM) in a temperature ramp^40^. An apparent melting temperature (Tm) can be obtained as a parameter of thermostability^32^ at the point where the unfolding rate is maximal. The CPM assay can be used to evaluate the folding state and stability of membrane proteins under different conditions^32,41,42^. In addition, shifts in the thermostability profiles provide information on the binding of inhibitors^17,21,32,43^ and other effectors^44^. Recently, it was demonstrated that shifts can also occur upon binding of substrates to transport proteins^32,45^, because specific interactions are formed between the protein and substrate molecules^45^. In principle, disruption of these interactions through mutations would lead to a decrease in the substrate-induced thermostability shift, providing a way to identify residues directly involved in substrate binding.

**Fig. 3:**
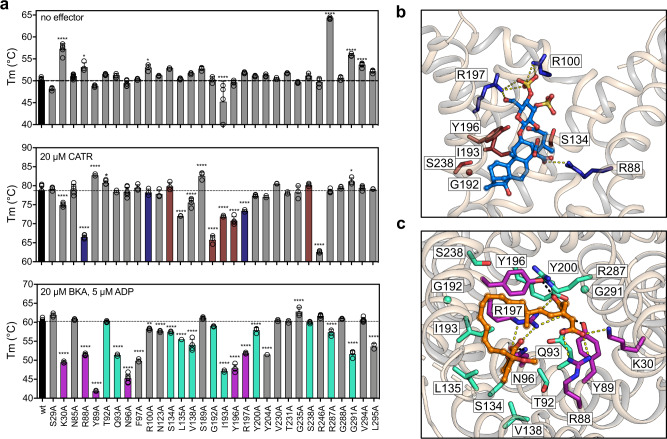
Mapping the binding sites of the specific inhibitors CATR and BKA. **a** CPM thermostability data of each protein in absence of effectors (top), or in presence of CATR (middle) or BKA (bottom). The bars and error bars represent the mean and sample standard deviation of eight independent experiments for the wild type and 2–7 for the variants. Significance analysis for each condition was performed with one-way ANOVA, as described in “Methods”. Only the significant are indicated (*p* > 0.01, ns; *p* ≤ 0.01, *; *p* ≤ 0.001, **; *p* ≤ 0.0001, ***; *p* ≤ 0.00001, ****). **b** Lateral view of ScAac2 cytoplasmic-open state structure in complex with CATR (PDB code: 4c9h chain A). The interacting residues of ScAac2 are conserved in TtAac, with the exception of K104 which is R100 in TtAac and the two proteins share 74% sequence identity. To facilitate comparison, we used the TtAac labelling (Supplementary Fig. 3). Residues that form ionic interactions (yellow dashes) and hydrophobic contacts with CATR (marine) are shown in dark blue and brown, respectively. Residue K104 of ScAac2 has been modelled to Arg (R100) for TtAac. **c** Matrix view of the matrix-open structure of TtAac with BKA bound (PDB code: 6gci chain A). Residues that form ionic interactions (yellow dashes) or hydrogen bonds (black dashes) with BKA (orange) are shown in purple, while residues that form hydrophobic contacts are shown in cyan. Source data for this figure are provided as a Source Data file.

To conduct the thermostability shift experiments we used purified proteins, expressed in the W303-1B yeast strain, which allowed the overexpression of all 36 variants, irrespective of their functional activity. All variants were expressed to similar levels and were correctly targeted to mitochondria. They were successfully purified using nickel affinity binding and on-column affinity tag cleavage (Supplementary Fig. 4). Their folding state and structural integrity were assessed with CPM assays in the presence and absence of the specific inhibitors CATR and BKA^17,21,32^ (Fig. 3). The stability of most mutants was not affected by the individual mutations, as their apparent Tm was not significantly different (48.2–52.8 °C) from the wild type (50.2 ± 0.6 °C) (Fig. 3a). The exceptions were variants L41A, Q44A, T146A, Y239A and N284A, which were in an unfolded state, as judged by the high initial fluorescence and the absence of a sigmoidal unfolding curve (Supplementary Fig. 5a). Notably, all of these residues are located in the matrix gate (Supplementary Fig. 5b, c). The matrix gate provides an insulation layer, preventing the dissipation of the proton motive force through proton leak, when the carrier is in the cytoplasmic-open state^17^. Furthermore, residue Q44 is the glutamine brace between H1 and H5^16^.

Importantly, all 31 folded variants, starting from the unliganded state, could bind both inhibitors, as is evident from the shifts to higher apparent Tm values, confirming that the unliganded carriers were folded. Only when the mutated residue participates in specific interactions with the inhibitor is a reduction or abolishment of the thermal shift observed. In this way, it was possible to map the binding sites of the inhibitors (Fig. 3b, c), which agreed well with most observed interactions in the structures of the CATR- and BKA-bound states^15–17^. The agreement is remarkable, given the fact that the thermostability data are measured with carriers in solution at temperatures in the 25–90 °C range, whereas crystal structures are determined in liquid nitrogen at −196 °C. Some apparent discrepancies require further comment. The bovine structure^15^ shows that the equivalent residues of K30 and R246 interact with CATR via ordered waters, agreeing with the reduced shifts observed for the relevant alanine variants (Fig. 3b). Residues L135 and V138 might form van der Waals contacts with the isovaleric group of CATR. In case of BKA, mutants F97A, R100A, N123A, Y204A and L295A presented a significantly reduced shift, even though these residues do not directly participate in its binding. They are part of the tyrosine braces and hydrophobic plug of the matrix-open state^17^, and thus are critical for its formation, a requirement for the binding of BKA. A similar observation was made for the residues comprising the cytoplasmic network^21^. Overall, the inhibitor analysis provided proof of principle that this assay can be used to detect residues involved in protein-ligand interactions. Furthermore, the thermostability data collectively proved that, apart from five variants (Supplementary Fig. 5), all unliganded proteins were well-folded and suitable for substrate-binding analyses.

We then determined an appropriate concentration range to characterize the substrate-induced thermostability shifts using the unliganded carriers. The shifts were expressed as a ΔTm value, calculated by subtracting the apparent, protein-specific, Tm in the absence of substrate from the apparent Tm at each substrate concentration. In agreement with previous work^45^, the ΔTm values of the wild type increased in a concentration-dependent manner, until saturation at 12 mM (ΔTm = 8.9 ± 1.8 °C) (Supplementary Fig. 6). Based on this titration experiment, five representative concentrations of ADP and ATP (0.1, 0.5, 1, 5, 10 mM) were selected to test the response of the variants. We note that these measurements do not provide a kinetic assessment, because the on and off rates of the substrate increase with increasing temperatures and because the protein unfolding and labelling are irreversible. The plateau occurs because substrate binding cannot prevent unfolding at these elevated temperatures.

### Five positively charged residues are critical for substrate binding

**Fig. 4:**
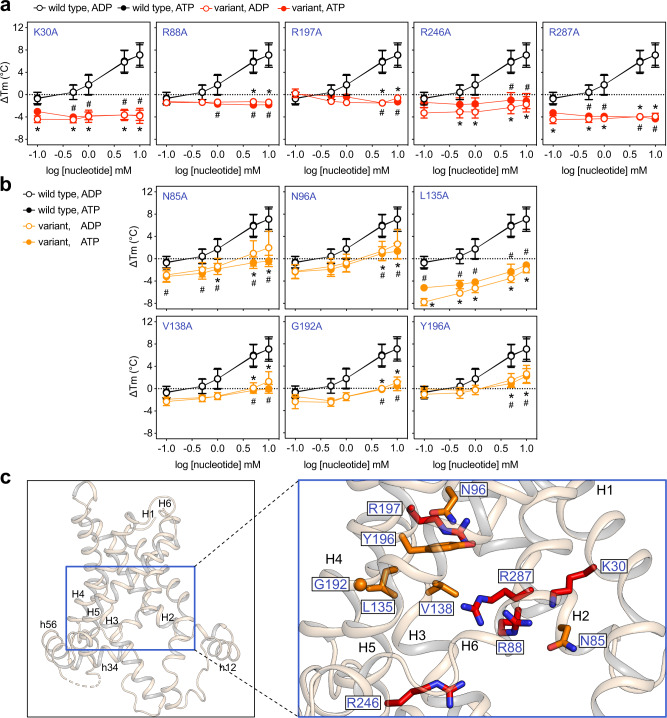
Five positively charged residues provide critical binding interactions and another six make important contributions. **a**, **b** Thermostability shift values (ΔTm) determined at 0.1, 0.5, 1, 5, 10 mM substrate concentration for the wild type and single alanine replacement variants. Circles and error bars represent the mean and standard deviation of eight independent experiments for the wild type and 3–7 for the variants. Empty and filled circles represent ADP and ATP, respectively. Data for the wild-type protein are shown in black, whereas those for the variants are shown in red (**a**) or in orange (**b**) for the critical and important residues, respectively. Note that for several data points the mean value and error bars of ADP vs ATP groups are identical and consequently their graphical representation is superimposed, with the ADP marker on top. Error bars are sometimes masked by the symbol. Significant differences were evaluated by two-way ANOVA with interaction, as described in “Methods”. Significant values (*p* ≤ 0.01) are indicated by * or # for ADP and ATP, respectively. **c** TtAac matrix-open BKA-bound structure (PDB code: 6gci chain A) (left) and close-up view (right) showing the analysed residues in red or orange stick and sphere for Gly representations. Helices are shown in wheat cartoon representation and are labelled. Source data for this figure are provided as a Source Data file.

**Fig. 5:**
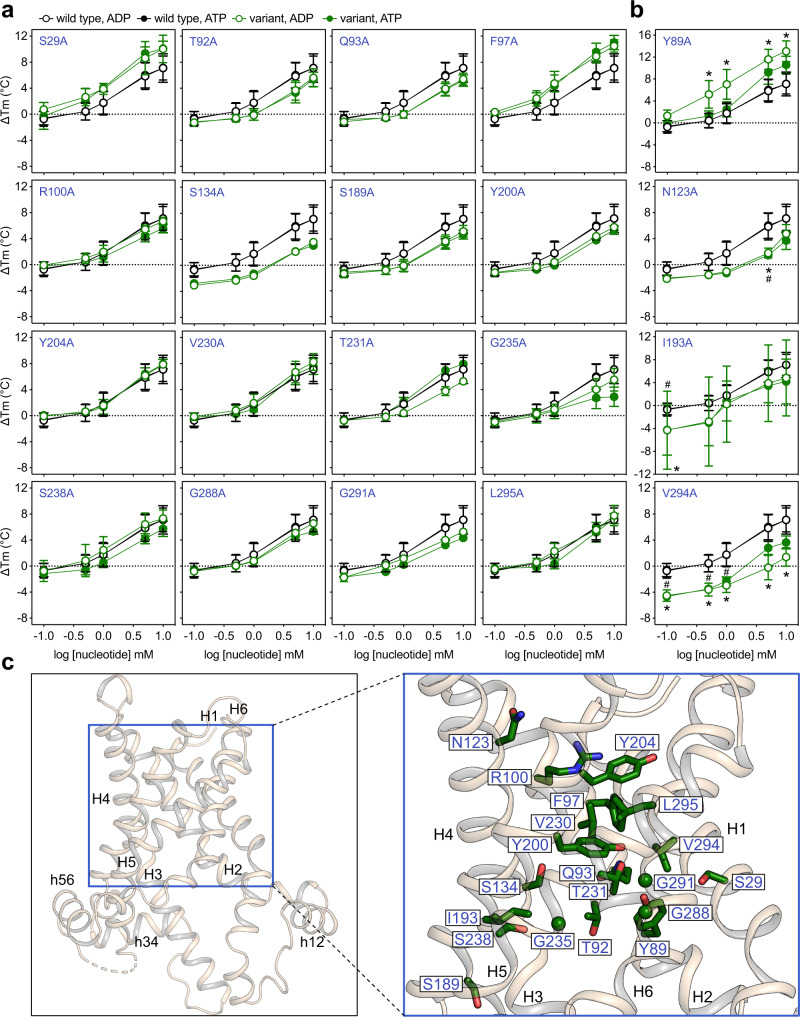
Substrate binding is confined to a small area of the translocation pathway. **a**, **b** Thermostability shift values (ΔTm) determined at 0.1, 0.5, 1, 5, 10 mM substrate concentration for the wild type and single alanine replacement variants. Circles and error bars represent the mean and standard deviation of eight independent experiments for the wild type and 3–5 for the variants. Empty and filled circles represent ADP and ATP, respectively. Data for the wild-type protein are shown in black, whereas the variant data are shown in green. Note that in several occasions the mean value and error bars of ADP vs ATP groups are identical and consequently their graphical representation is superimposed, with ADP marker on top. Error bars are smaller than the symbol whenever not shown. The statistical evaluation was carried out as described in “Methods”. Significant values (*p* ≤ 0.01) are indicated by * or # for ADP and ATP, respectively. **c** TtAac matrix-open BKA-bound structure (PDB code: 6gci chain A) (left) and close-up view (right) showing the analysed residues in green stick and sphere for Gly representations. Helices are shown in wheat cartoon representation and are labelled. Source data for this figure are provided as a Source Data file.

We subsequently measured the thermostability shifts in the presence of ADP and ATP for all 31 folded variants. With respect to their response to substrate, they were categorised in three classes (Figs. 4, 5). In the first class, variants K30A, R88A, R197A, R246A and R287A showed no concentration-dependent thermostability shift in the presence of either substrate (Fig. 4a), indicating that each of these residues forms a critical interaction with the substrate. The variants consistently showed no growth in complementation assays (Fig. 2a, Supplementary Fig. 2). These positively charged residues are found in the middle of the water-filled cavity (Fig. 4c) and their alanine replacement results in loss of a positive charge.

### Six polar, aliphatic and aromatic residues make significant contributions to substrate binding

The second class of alanine-replacement variants involved six residues (N85, N96, L135, V138, G192 and Y196), which were in the vicinity of the five critical residues and had significantly altered thermostability profiles compared to the wild type (Fig. 4b). These variants did show concentration-dependent shifts in presence of substrate, but they were significantly smaller than those of the wild-type protein. Significant differences were mostly obtained for the two highest concentrations tested, for both ADP and ATP (Fig. 4b). Since the substrate-induced shifts were not completely abolished, each residue makes a contribution to substrate binding. In support of their contributing role, variants L135A, G192A and Y196A did not grow in the complementation study at all (<3%), whereas N85A, N96A and V138A grew significantly less (42 ± 23%, 18 ± 10% and 53 ± 14% respectively) (Fig. 2a, Supplementary Fig. 2). Notably, alanine replacement did not cause protein instability or disorder of the binding pocket (Fig. 3) for any of the eleven variants in these two classes, emphasising that the observed effects are indeed specific for substrate binding.

### Substrate binding is confined to a single area of the translocation pathway

Importantly, the third class of variants involved 20 of the 31 variants, which presented substrate-induced shifts that were not significantly different from those of the wild-type protein (Fig. 5). The thermostability shifts of mutant proteins S29A, V230A, G288A and G291A were not different from the wild type at any concentration of ADP and ATP (Fig. 5a), and these variants were fully functional (Fig. 2a, Supplementary Fig. 2). Although variants F97A, S189A, Y204, G235A and L295A did not complement growth and T92A, Q93A, R100A, S134, Y200A, T231A and S238A complemented only partially (Fig. 2a, Supplementary Fig. 2), they all had similar thermostability shifts as the wild-type protein in the presence of substrates (Fig. 5a). These results demonstrate that these mutations affected important steps of the transport mechanism, but not substrate binding.

Four variants presented some differences from the wild type, but not consistently. Variant Y89A could complement growth partially (36 ± 19%) (Fig. 2a), but exhibited greater shifts than the wild type for both nucleotides (significant only for ADP) (Fig. 5b), indicating that this residue is not directly involved in substrate binding. Variant N123A was partially active (50 ± 9%) (Fig. 2a) and the substrate-induced shifts were significantly different from the wild type only at 5 mM (Fig. 5b). The strain expressing variant V294 grew on glycerol to wild-type levels (84 ± 14%) (Fig. 2a) and the purified protein was more stable (53.7 ± 0.5 °C) than the wild type (50.2 ± 0.6 °C) (Fig. 3a). This resulted in spurious significance at low substrate concentrations, but it reached wild-type levels at the highest concentration for ATP (Fig. 5b). Finally, the stability of mutant protein I193A was highly variable (Figs. 5b, 3a), precluding an assessment of the significance of the substrate-induced shifts.

Residues that are not involved in substrate binding were located throughout the translocation pathway, but also in close proximity to the ones that were shown to participate in substrate binding (Fig. 5c). These results show that the thermostability shift assays can accurately identify individual residues participating in protein-substrate interactions and that substrate binding is confined to a cluster of residues in the translocation pathway. Remarkably, for all tested proteins, there were no significant differences between the thermal shifts induced by ADP or ATP at any substrate concentration (Supplementary Fig. 7).

## Discussion

The mitochondrial ADP/ATP carriers have unique features that enable them to achieve a high flux of ADP and ATP across the mitochondrial inner membrane to sustain our daily activities. Furthermore, they have evolved to navigate the challenge of importing negatively charged ADP against the large electrochemical potential generated across the mitochondrial inner membrane by the respiratory chain. Despite being studied for more than 55 years, there are still many open questions about the transport mechanism, in particular about substrate binding and its effect on the interconversion between the matrix- and cytoplasmic-open states.

**Fig. 6:**
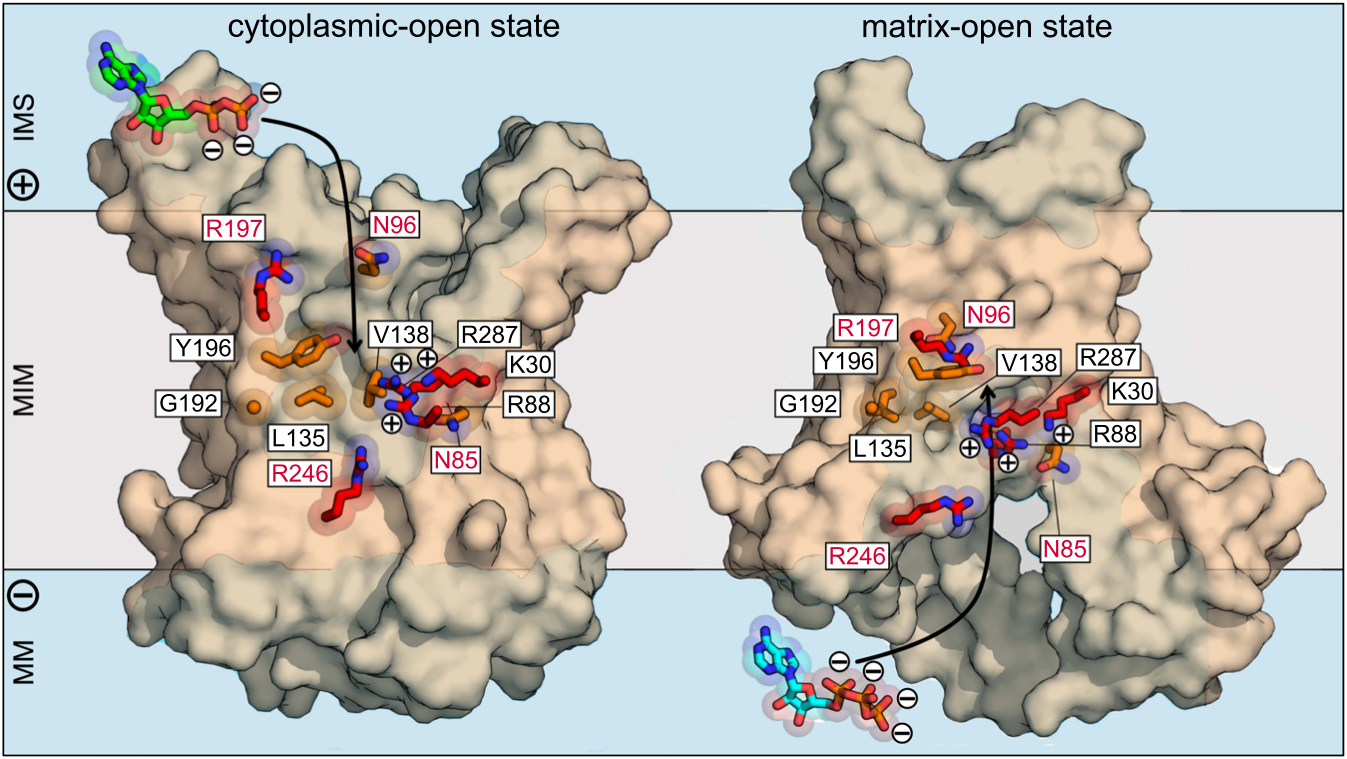
Two asparagine/arginine pairs guide substrates ADP and ATP to and away from the central binding site as part of the transport cycle. Lateral views from the membrane of the cytoplasmic-open state (left) (ScAac2, PDB code 4c9h chain A) and matrix-open state (PDB code 6gci chain A) (right) of the mitochondrial ADP/ATP carrier. The interacting residues of ScAac2 are conserved in TtAac and to facilitate comparison we used the TtAac labelling (Supplementary Fig. 3). The water-accessible surfaces, determined by HOLE^59^, are shown in transparent blue. Residues of the water-filled cavity that have critical and important roles in substrate binding are represented in red and orange, respectively, while the substrates ADP and ATP are shown in green and cyan, respectively. The water-accessible surfaces are shown in blue.

Many proposals have been made for the location of the substrate-binding site^19,20,24–31^, but here we address this question experimentally with the relevant substrates. Our approach allows a complete assessment of the substrate-binding process, highlighting only significant interactions. Having investigated all residues of the translocation pathway between the two salt bridge networks, we have identified those with critical and contributing roles in substrate binding and have shown that they cluster together, approximately halfway along the translocation pathway. The observation that a single mutation can abolish binding altogether indicates that there are no other significant substrate-binding sites elsewhere, such as the matrix loops or C-terminal region, as claimed previously^24–27^. It is possible that other observed interactions^28–31^ are transient, including those of the “tyrosine ladder”^15,28^, for which we find no experimental support. Furthermore, since the thermostability shifts induced by ADP and ATP were not statistically different from each other at any concentration for any of the tested proteins (Supplementary Fig. 7), both substrates must bind to the same set of residues in a similar way (Fig. 6). Given that ADP/ATP carriers function as monomers^14,17,46–50^, the import and export steps must therefore be consecutive, implying a ping-pong kinetic mechanism.

In the case of positively charged residues K30, R88, R197, R246 and R287, individual alanine replacements led to the loss of function (Fig. 2a) and complete abolishment of the substrate-induced thermostability shifts (Fig. 4a). Given their chemical properties, these residues can be engaging in ionic interactions with the negatively charged phosphate groups of the nucleotides. In this way, they make relatively large contributions to the overall interaction energy of substrate binding, which facilitates the conformational changes required for transport^22,23^. On the other hand, alanine replacement of residues N85, N96, L135, V138, G192 and Y196 resulted in partial or complete loss of function (Fig. 2a) and in significantly reduced thermostability shifts in the presence of substrates (Fig. 4b). Given the chemical properties and relative positions of these residues, they are likely to be jointly involved in binding of the relatively hydrophobic adenosine moiety, forming weaker interactions than the aforementioned charged residues, i.e., hydrophobic and aromatic stacking interactions. Thus, they will make smaller individual contributions to the overall interaction energy of substrate binding.

Earlier sequence analyses, which considered the chemical properties of substrates and distance constraints, proposed that there are three ‘contact points’ involved in substrate binding, which are universal to all members of the SLC25 family and are found on the even-numbered transmembrane helices^18,19^. Later, it was discovered that they also form hinge points between the core and the gate elements of the protein domains, thus linking substrate binding to a common transport mechanism^4,5,17^. The critical residues identified in this study, R88 and R287, on H2 and H6 respectively, correspond to contact points I and III. Critical residue K30 on H1 is located between them at the same height in the central cavity. Residues that make an important contribution to binding, G192 and Y196 on H4, are in contact point II, whereas L135 and V138 are nearby. All these seven residues are accessible in both conformational states and have similar conformers (Fig. 6), showing that they participate jointly in substrate binding, forming the main substrate-binding site throughout the conformational changes. The central location of this site is consistent with the formation and disruption of the matrix and cytoplasmic gates on either side, as the carrier switches between states^4,5,17^. In addition, it was also shown to be the fulcrum of all conformational changes that occur during the transport cycle^17^.

The remaining four residues form two asparagine/arginine pairs, N85/R246 and N96/R197, which are located on opposite sides of the main site. They have the same chemical properties and thus could fulfil similar roles. Comparing the cytoplasmic- and matrix-open structures clearly shows that these Asn/Arg pairs change conformers in a state-dependent manner (Fig. 6). In the cytoplasmic-open state, residues N96/R197 point towards the opening of the cavity, whereas they are part of the main binding site in the matrix-open state. Conversely, residues N85/R246 are pointing towards the opening of the cavity in the matrix-open state, whereas they are part of the main binding site in the cytoplasmic-open state (Fig. 6). Since these pairs point to the opening of the cavity, they are likely to be responsible for the initial binding and final release of the nucleotides.

**Fig. 7:**
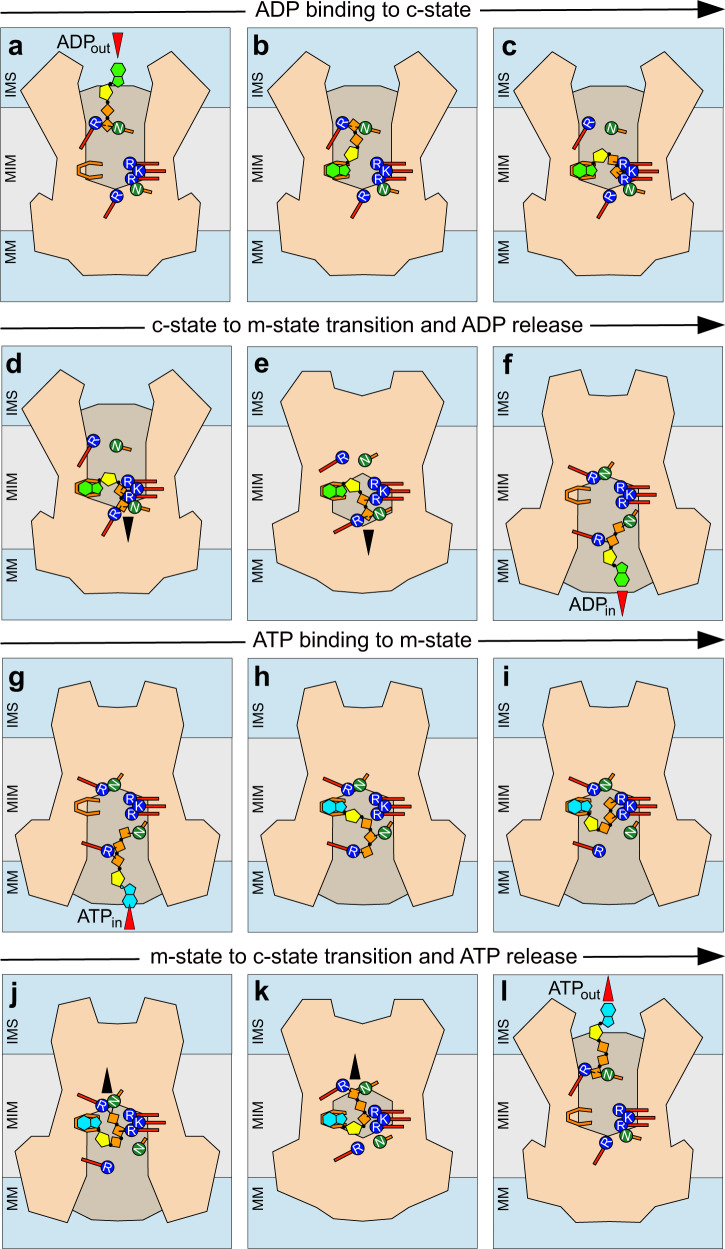
Schematic representation of the substrate-binding events as part of the transport cycle. Different stages of the transport cycle, **a**–**d**, **l** cytoplasmic-open state, **e**, **k** occluded state, and **f**–**j** matrix-open state. The substrates ADP and ATP have an adenine moiety (green and cyan, respectively), a ribose moiety (yellow) and two or three phosphate moieties, respectively (orange). The adenosine binding site is represented by a horseshoe shape, whereas positively charged and polar residues of the binding site are shown in blue and green, respectively. The black arrowheads show the substrate movements that induce the conversion between states, whereas the red ones indicate entry and exit of the substrates.

In summary, there is a single binding site for the adenosine moiety and several positively charged and polar residues for binding of the phosphate moieties, which engage in a state-dependent or state-independent manner, indicating a step-wise process. Their role in the transport cycle can be explained in a stepwise mechanism, which is our current model of the substrate-binding events. First, ADP enters the cavity of the carrier in the cytoplasmic state by Brownian motion and electrostatic attraction^28–30^, and the phosphate moieties engage with the N96/R197 pair (Fig. 7a). Once bound, the hydrophobic adenine moiety of ADP is free to bind to the hydrophobic/aromatic binding site (G192, Y196, L135 and V138) for substrate recognition, shielding it from the water phase (Fig. 7b)^18,19,51^. This is an important step, as mitochondrial ADP/ATP carriers have a narrow specificity, transporting only ADP, ATP and their deoxy-variants^30,51^. Next, the negatively charged phosphate moieties bind to the positively charged K30, R88, and R287 of the main binding site, positioning the ADP molecule in the central area and neutralising its charge (Fig. 7c). All contact points of the binding site are now engaged, allowing progression to the next stages. The terminal phosphate of ADP starts to engage with the N85/R246 pair (Fig. 7d), continuing the conformational changes to the occluded (Fig. 7e) and matrix-open states (Fig. 7f). A similar, but not identical, pose as in Fig. 7d has been observed in MD simulations^28,30^, but the simulation times (<1 μs) were too short for completion of the half cycle (~0.5 ms). Finally, the N85/R246 pair brings ADP closer to the mouth of the cavity and releases it into the mitochondrial matrix for ATP synthesis (Fig. 7f). The newly synthesized ATP is transported out of the mitochondrion by the carrier in a similar series of interactions and conformational changes^4,5,17^, but occuring in reverse (Fig. 7g-l).

In this step-wise manner, the large and conformationally diverse ADP and ATP molecules are directed through a series of conformers that accompany the complex conformational changes required for their translocation. Furthermore, the position of the central site with the two Asn/Arg pairs on either side indicates that the substrates are put through a similar set of interactions independent of the direction from which they came, explaining the observed reversible nature of transport. In agreement with previous observations, the direction of transport is not determined by the protein properties, but by the chemical gradients of the substrates and the membrane potential.

Moreover, the electrostatic properties of the identified binding residues can explain an important aspect of the bioenergetics of adenine nucleotide transport in mitochondria. During the import of ADP three negative charges are moved against the membrane potential, whereas during export of ATP four negative charges are moved with it. The charge movements have been measured directly using caged nucleotides, giving values of +0.3 or +0.5 for the ADP import step and −0.7 or −0.5 for the ATP export step^52,53^. Based on these observations, it was proposed that the substrate-binding site should contain 3.3 or 3.5 positive counter charges^52,53^. These measurements agree well with the three positive charges of K30, R88 and R287 in the main binding site, which reorient with the substrate to the other compartment, as the carrier changes conformation. The partial charges could be due to the state-dependent interactions of either R197 or R246, which are transient and weaker in nature. The partial charges stimulate both the import of ADP and the export of ATP in the presence of a large membrane potential and minimise the electrophoretic force on the bound substrates in the occluded state. The observed equimolar exchange of ADP and ATP is not determined by the properties of the substrate-binding site, but by the two salt bridge networks, which prevent the conformational changes in the absence of substrate. Only substrate binding will lead to a conformational change, as it provides energy input to break the network^22,23^, and thus there will be one for one exchange of the two substrates.

Interestingly, variants K30A, R88A and R287A were more thermostable (57.3 ± 1.2, 53.1 ± 1, 64.4 ± 0.2 °C respectively) than the wild-type carrier (50.2 ± 0.6 °C) (Fig. 3), due to the removal of one of the positive charges that are located close together in the central cavity. Thus, the carrier might be prevented from interconverting between states in the absence of substrates, because of the high energy barrier imposed by the repulsion of these three positive charges as the carrier changes conformation. Binding of the negatively charged ADP and ATP to these residues would lower the free energy by neutralising them, leading to substrate translocation. The G291A and V294A mutations, which affect interhelical residues^17^, may impede the dynamics, whereas R100A, which is equivalent to a tyrosine brace^17^, may weaken the cytoplasmic network, making the cytoplasmic-open state stochastically more likely. Thus, these mutations also make the interconversion of states less favourable, leading to more stable variants (Fig. 3).

In conclusion, the properties of the identified binding site explain many features of the mechanism that heretofore had no molecular explanation. The same principles, as discovered here, could apply to the binding and transport of substrates for other members of the SLC25 mitochondrial carrier family, which are currently under investigation.

## Methods

### Construction of expression strains

The subject of the study was the mitochondrial ADP/ATP carrier of *Thermothelomyces thermophila*. For expression of the wild-type mitochondrial ADP/ATP carrier, the gene was codon-optimised and cloned in a pYES3/CT derivative vector (Carlsbad, CA, Invitrogen, USA), containing the constitutively active pPIC2 promoter of the phosphate carrier *pic2* of *Saccharomyces cerevisiae*^21,46^. Single alanine replacements were introduced in the indicated positions for each variant by replacing the relevant codon with GCT (the most frequent codon for alanine in the yeast genome), using overlap-extension PCR^54^ with KOD HotStart polymerase (Novagen). The primers to introduce the mutations are shown in Supplementary Table 1. The expression plasmids were transformed into the *S. cerevisiae* strains WB-12 (MATα *ade2-1 trp1-1 ura3-1 can1-100 aac1::LEU2 aac2::HIS3*)^33^, in which aac1 and aac2 genes are disrupted and in strain W303-1B (MATα *leu2‐3, 112 trp1‐1 can1‐100 ura3‐1 ade2‐1 his3‐11,15*), using the LiAc/SS carrier DNA/PEG method^55^. Successful transformants were selected on synthetic‐complete tryptophan‐dropout medium (Formedium) plates, supplemented with 2% (w/v) glucose. An empty vector, generated by restoring the multiple cloning site of the vector, was used as control.

### Functional complementation assays and densitometry analysis

The WB-12 strain expressing the wild-type or mutant proteins or the empty vector was grown overnight in synthetic‐complete tryptophan‐dropout medium supplemented with 2% (w/v) glucose. Cells were washed three times with ultra-pure sterile water, so that glucose was completely removed and diluted to an OD_600_ of 1. Four serial dilutions (1:10) were made and 3.5 μL of the starting culture and each dilution were dispensed on a YPG plate and incubated at 30 °C for 72 h. Only the second and third dilutions were used for the analysis. Each plate contained the wild type and empty vector control. Growth of the wild type and alanine replacement mutants was quantified by densitometry. Scanned images or photographs of the plates were analysed using the Fiji software (version 2.3.0/1.53q)^56^, specifically the ‘Gel analysis’ tool. The density of the colonies (whole surface) was measured and was expressed as a percentage of the density of the wild type of the same plate and dilution, after subtracting the density of the empty vector from both. The growth percentages calculated from the second and third dilutions (10^−2^, 10^−3^) were averaged and this value constituted one biological repeat. The experiments were repeated independently four times.

### Large-scale expression and mitochondrial preparations

For each wild-type and variant protein, a 1-L pre-culture of the expression strain was used to inoculate 10-L of YPG medium plus 0.1% (w/v) glucose in an Applikon Autoclavable 15 L Bio-bundle with eZ control. Either the WB-12 or W303-1B strain was used as the genetic background for the wild type, as there were no observed differences in the yield, stability or activity of the carrier purified from either strain. For the variants, the W303-1B strain was selected, as this strain allowed for sufficient expression, irrespective of the functional state of the mutant protein. Cells were lysed using a DYNO-MILL (Willy A. Bachofen) and mitochondria were isolated^57^, snap-frozen in liquid nitrogen and stored at −70 °C until use.

### Protein purification

Isolated yeast mitochondria (~350 mg total protein) were solubilised by resuspension in a buffer containing 20 mM Tris–HCl pH 7.5, 10% (v/v) glycerol, 150 mM NaCl, 20 mM imidazole pH 7.5, one Complete EDTA‐free protease inhibitor cocktail tablet (Roche) and 1% (w/v) dodecyl-β-maltoside (Glycon Biochemicals GmbH) followed by gentle agitation at 4 °C for 1 h. Particulate material was removed by ultracentrifugation (235,000 × *g*, 45 min, 4 °C) and the soluble fraction was incubated with previously washed and equilibrated (20 mM Tris-HCl pH 7.5, 150 mM NaCl) nickel Sepharose beads (GE Healthcare) for 2 h, at 4 °C, under gentle agitation. Unbound proteins were removed by centrifugation (200 × *g*, 5 min, 4 °C) and the bound fraction was placed in an empty column (BioRad), where it was washed with 40 column volumes of Buffer A (20 mM HEPES-NaOH pH 7.5, 150 mM NaCl, 20 mM imidazole pH 7.5, 0.1% (w/v) dodecyl-β-maltoside, 0.1 mg/mL tetraoleoyl cardiolipin), followed by 25 column volumes of Buffer B (20 mM HEPES-NaOH pH 7.5, 150 mM NaCl, 0.1% (w/v) dodecyl-β-maltoside, 0.1 mg/mL tetraoleoyl cardiolipin). The column material was then resuspended with 500 μL Buffer B and supplemented with 5 mM CaCl_2_ and 10 μg Factor Xa protease (New England Biolabs) for on-column digestion, overnight at 10 °C, with gentle agitation. For variants N123A, G192A, I193A, R197A and R246A the on-column digestion step was reduced to 2 h, using 30 μg Factor Xa protease, due to protein instability over time. Following Factor Xa treatment, the cleaved protein was separated from the nickel Sepharose resin with an empty Proteus Midi spin column (Generon) (200 × *g*, 5 min, 4 °C). Protein concentrations were measured with a spectrophotometer (NanoDrop Technologies) at 280 nm or the Bicinchoninic acid (BCA) protein assay kit (Thermo Fisher Scientific). Freshly purified protein was used for the CPM thermostability shift assays.

### CPM thermostability shift assays

The assessment of protein population stability for the wild type and variants in the presence and absence of effectors (substrates or inhibitors) was performed via thermal unfolding, induced by a temperature ramp^32^. In this assay, initially inaccessible cysteine residues become solvent exposed through thermal denaturation and react with the fluorophore N‐[4‐(7‐diethylamino‐4‐methyl‐3‐coumarinyl)phenyl]‐maleimide (CPM)^40^. The reaction leads to the formation of fluorescent adducts, which is monitored by using a rotary quantitative PCR (qPCR) instrument (Rotor gene Q, Qiagen). For each experiment, 5 mg/mL CPM stock solution in dimethyl sulfoxide was diluted to 0.1 mg/mL in purification Buffer B (20 mM HEPES–NaOH pH 7.5, 150 mM NaCl, 0.1% (w/v) dodecyl-β-maltoside, 0.1 mg/mL tetraoleoyl cardiolipin) and was equilibrated in the dark, for 10 min, at room temperature. Three micrograms of purified protein were mixed with the relevant effector (where indicated) and diluted into Buffer B to a final volume of 45 μL, to which 5 μL of CPM solution of 0.1 mg/mL were added. The effectors ADP and ATP were added at final concentrations of 0.1, 0.5, 1, 5, and 10 mM, CATR at 20 μM and BKA at 20 μM plus 5 μΜ ADP, which was added to allow the carriers to cycle between states in order to bind the inhibitor^21^. The mixture was vortexed very briefly and was incubated in the dark, for 10 min, at 4 °C, before being placed in the qPCR instrument. Subsequently, the protein population was assayed in a temperature gradient from 25 to 90 °C, corresponding to a rate of ~4 °C per min. The increasing fluorescence emitted by the protein-fluorophore adduct was measured in the HRM channel of the machine (excitation at 440–480 nm and emission at 505–515 nm). Unfolding profiles were analysed with the Rotor‐Gene Q software 2.3, where the inflection point of the unfolding curves was used to determine the apparent melting temperature (Tm) of the proteins in the different conditions tested. A ΔTm value was obtained by subtracting the Tm of the protein in absence of effectors from the Tm at each concentration of the effectors.

#### Model building

The model of TtAac in the cytoplasmic-open state was generated with Modeller version 9.22^58^, using the structure-based alignments of chains A of PDB codes 1okc, 4c9h, 4c9q, and 4c9j and the restraints that residues 140 to 156 should be helical as in the matrix-open state structure of TtAac, PDB code: 6gci. The DOPE scoring was used to check out the models. The clash score was improved by energy minimising the structure in Chimera. After inspection of the binding site residues, K30 had shifted position compared to the equivalent residue in 4c9h, and was returned to the original conformer. Finally, the N- and C-termini were truncated to reflect the structure in 4c9h. The water-accessible surfaces were determined by HOLE^59^.

### Statistical analysis

Results in all figures are shown as mean ± SD of the indicated number of experiments. Statistical analysis of the data was performed with GraphPad Prism version 9.2.0 (GraphPad Software, San Diego, California USA) as described below. In the complementation experiments, growth differences were analysed by a two-tailed one-sample t-test, comparing the growth percentage of the variants to the mean percentage growth of the wild type. Differences between the variant-specific Tm values vs the wild-type Tm value were evaluated with one-way ANOVA, followed by a Dunnett post hoc test to correct for multiple comparisons. Differences between the variant-specific Tm values vs the wild-type Tm in the presence of inhibitors (CATR and BKA) was assessed in the same way (one-way ANOVA, followed by Dunnett post hoc test). Regarding the thermal shifts induced by substrate (ADP or ATP) on protein variants, differences between groups were assessed by a two-way ANOVA with interaction for each nucleotide concentration tested. Simple effects between protein variants within the substrate level were analysed and corrected using the Dunnet post hoc test against the wild-type protein (visualised in Figs. 4, 5). Simple effects between substrates for each protein level were analysed and corrected by Sidak post hoc test (visualised for 10 mM in Supplementary Fig. 7). Differences were considered significant at the 1% level.

### Reporting summary

Further information on research design is available in the Nature Research Reporting Summary linked to this article.

## Supplementary information

Supplementary Information

Reporting Summary

## Data Availability

Source Data are provided with this paper. All complementation and thermostability shift data generated in this study have been deposited in the Mendeley database under accession code 10.17632/mrhnw45w5y.1 (https://data.mendeley.com/datasets/mrhnw45w5y/1) Source data are provided with this paper.

## Source data

Source Data
