# Supplementary material for: Substrate binding in the mitochondrial ADP/ATP carrier is a step-wise process guiding the structural changes in the transport cycle
Source: Nat Commun. 2022 Jun 23;13:3585. doi: 10.1038/s41467-022-31366-5 (PMC9226169; doi:10.1038/s41467-022-31366-5)
Supplement: Supplementary file 1 — Supplementary Information [file 41467_2022_31366_MOESM1_ESM.pdf]

Supplementary Information

Identification of a single substrate binding site for ADP and ATP in the central cavity of the mitochondrial ADP/ATP carrier

Vasiliki Mavridou, Martin S. King, Sotiria Tavoulari, Jonathan J. Ruprecht, Shane M. Palmer and Edmund R.S. Kunji

Medical Research Council Mitochondrial Biology Unit, University of Cambridge, Cambridge Biomedical Campus, Keith Peters Building, Hills Road, Cambridge, CB2 0XY, United Kingdom,

Supplementary Table 1 | DNA oligonucleotide sequences for PCR primers.

Oligonucleotide	Sequence 5'-3'
TtAac no tag forward	CATGACATGTCTAAACAAGAAACTAAAATTTTAGGAATGCCACCCTTCGTG
TtAac no tag reverse	CTAGCTCGAGCTATCATTAACCAGATCCACCTTTAAAAGCTTTGCCGAATAGTAGAATTTGC
TtAac His tag Factor Xa forward	GACTCATTGACAGTTGTAAAGCCATGGCTCATCATCACCATCACCAT
TtAac His tag Factor Xa reverse	TCTAGACTCGAGTCTAGATCATTTGCCGAATAGTAGAATTTGCAATTGATCG
S29A forward	GTGGTGTTTCTGCAGCAGTTGCTAAAACAGCTGCTGCTCCAAT
S29A reverse	ATTGGAGCAGCAGCTGTTTTAGCAACTGCTGCAGAAACACCAC
K30A forward	CTGCAGCAGTTAGTGCTACAGCTGC
K30A reverse	GCAGCTGTAGCACTAACTGCTGCAG
L41A forward	GCTGCTCCAATTGAAAGAATTAAGGCTTTGGTACAAAACCAAGATGAAATG
L41A reverse	CATTTCATCTTGGTTTTGTACCAAAGCCTTAATTCTTTCAATTGGAGCAGC
Q44 forward	AATTGAAAGAATTAAGTTGTTGGTAGCTAACCAAGATGAAATGATTAAGGCTG

Q44 reverse	CAGCCTTAATCATTTCATCTTGGTTAGCTACCAACAACTTAATTCTTTCAATT
N85A forward	TTATGGAGAGGTAATACTGCCGCTGTTATAAGATATTTTCCAACC
N85A reverse	GGTTGGAAAATATCTTATAACAGCGGCAGTATTACCTCTCCATAA
R88A forward	GCCAACGTTATAGCTTATTTTCCAACCCAG
R88A reverse	CTGGGTTGGAAAATAAGCTATAACGTTGGC
Y89A forward	ATACTGCCAACGTTATAAGAGCTTTTCCAACCCAGGCATTGAA
Y89A reverse	TTCAATGCCTGGGTTGGAAAAGCTCTTATAACGTTGGCAGTAT
T92A forward	CCAACGTTATAAGATATTTTCCAGCTCAGGCATTGAACTTTGCTTTTAG
T92A reverse	CTAAAAGCAAAGTTCAATGCCTGAGCTGGAAAATATCTTATAACGTTGG
Q93A forward	CGTTATAAGATATTTTCCAACCGCTGCATTGAACTTTGCTTTTAGAG
Q93A reverse	CTCTAAAAGCAAAGTTCAATGCAGCGGTTGGAAAATATCTTATAACG
N96A forward	GATATTTTCCAACCCAGGCATTGGCTTTTGCTTTTAGAGATAAGTTTAA
N96A reverse	TTAAACTTATCTCTAAAAGCAAAAGCCAATGCCTGGGTTGGAAAATATC
F97A forward	TTCCAACCCAGGCATTGAACGCTGCTTTTAGAGATAAGTTTAA
F97A reverse	TTAAACTTATCTCTAAAAGCAGCGTTCAATGCCTGGGTTGGAA
R100A forward	GAACTTTGCTTTTGCTGATAAGTTTAAGGC
R100A reverse	GCCTTAAACTTATCAGCAAAAGCAAAGTTC
N123A forward	ATGCCAAATGGATGGCAGGTGCTCTAGCCAGCGGTGGTGCAGC
N123A reverse	GCTGCACCACCGCTGGCTAGAGCACCTGCCATCCATTTGGCAT
S134 forward	GTGGTGCAGCTGGTGCCACCGCTTTGTTGTTTGTATACAGCTT
S134 reverse	AAGCTGTATACAAACAACAAAGCGGTGGCACCAGCTGCACCAC
L135A forward	GTGCAGCTGGTGCCACCTCGGCTTTGTTTGTATACAGCTTGGA
L135A reverse	TCCAAGCTGTATACAAACAAAGCCGAGGTGGCACCAGCTGCAC

V138A forward	GTGCCACCTCGTTGTTGTTTGCTTACAGCTTGGACTATGCAAG
V138A reverse	CTTGCATAGTCCAAGCTGTAAGCAAACAACAACGAGGTGGCAC
T146A forward	ACAGCTTGGACTATGCAAGAGCTAGATTGGCTAATGATGCAAA
T146A reverse	TTTGCATCATTAGCCAATCTAGCTCTTGCATAGTCCAAGCTGT
S189A forward	TGTACAGAGGTTTCGGTCCAGCTGTGGCCGGTATTGTTGTTTA
S189A reverse	TAAACAACAATACCGGCCACAGCTGGACCGAAACCTCTGTACA
G192A forward	GGTCCATCCGTGGCCGCTATTGTTGTTTACAGA
G192A reverse	TCTGTAAACAACAATAGCGGCCACGGATGGACC
I193A forward	CCATCCGTGGCCGGTGCTGTTGTTTACAGAGGT
I193A reverse	ACCTCTGTAAACAACAGCACCGGCCACGGATGG
Y196A forward	GCCGGTATTGTTGTTGCTAGAGGTTTATATTTC
Y196A reverse	GAAATATAAACCTCTAGCAACAACAATACCGGC
R197A forward	GGTATTGTTGTTTACGCTGGTTTATATTTCGGT
R197A reverse	ACCGAAATATAAACCAGCGTAAACAACAATACC
Y200A forward	GGTATTGTTGTTTACAGAGGTTTAGCTTTCGGTATGTATGACTCAATTAAG
Y200A reverse	CTTAATTGAGTCATACATACCGAAAGCTAAACCTCTGTAAACAACAATACC
Y204 forward	GAGGTTTATATTTCGGTATGGCTGACTCAATTAAGCCAGTCGT
Y204 reverse	ACGACTGGCTTAATTGAGTCAGCCATACCGAAATATAAACCTC
V230A forward	CCTTTCTTTTAGGTTGGTGTGCTACTACCGGTGCCGGTATTGC
V230A reverse	GCAATACCGGCACCGGTAGTAGCACACCAACCTAAAAGAAAGG
T231A forward	TTCTTTTAGGTTGGTGTGTTGCTACCGGTGCCGGTATTGCCTC
T231A reverse	GAGGCAATACCGGCACCGGTAGCAACACACCAACCTAAAAGAA
G235A forward	GGTGTGTTACTACCGGTGCCGCTATTGCCTCCTATCCATTAGA
G235A reverse	TCTAATGGATAGGAGGCAATAGCGGCACCGGTAGTAACACACC

S238A forward	GGTGCCGGTATTGCCGCTTATCCATTAGACACC
S238A reverse	GGTGTCTAATGGATAAGCGGCAATACCGGCACC
Y239A forward	CCGGTGCCGGTATTGCCTCCGCTCCATTAGACACCGTGAGAAG
Y239A reverse	CTTCTCACGGTGTCTAATGGAGCGGAGGCAATACCGGCACCGG
R246A forward	TTAGACACCGTGAGAGCTAGAATGATGATGACA
R246A reverse	TGTCATCATCATTCTAGCTCTCACGGTGTCTAA
N284 reverse	TCCCGGCTCGAGTCTAGATCATTTGCCGAATAGTAGAATTTGCAATTG ATCGTATATGGACAACACACCTGCACCAGCAACGCCTCTCAAGATAGC AGCACCAGCTCCCTTAA
R287A forward	GCTAATATCTTGGCTGGCGTTGCTGGTGCA
R287A reverse	TGCACCAGCAACGCCAGCCAAGATATTAGC
G288A reverse	TCCCGGCTCGAGTCTAGATCATTTGCCGAATAGTAGAATTTGCAATTG ATCGTATATGGACAACACACCTGCACCAGCAACAGCTCTCAAGATATT AGCAC
G291A reverse	TCCCGGCTCGAGTCTAGATCATTTGCCGAATAGTAGAATTTGCAATTG ATCGTATATGGACAACACACCTGCAGCAGCAACGCCTCTCAAGA
V249A reverse	TCCCGGCTCGAGTCTAGATCATTTGCCGAATAGTAGAATTTGCAATTG ATCGTATATGGACAAAGCACCTGCACCAGCAACGC
L295A reverse	TCCCGGCTCGAGTCTAGATCATTTGCCGAATAGTAGAATTTGCAATTG ATCGTATATGGAAGCCACACCTGCACCAGCAA

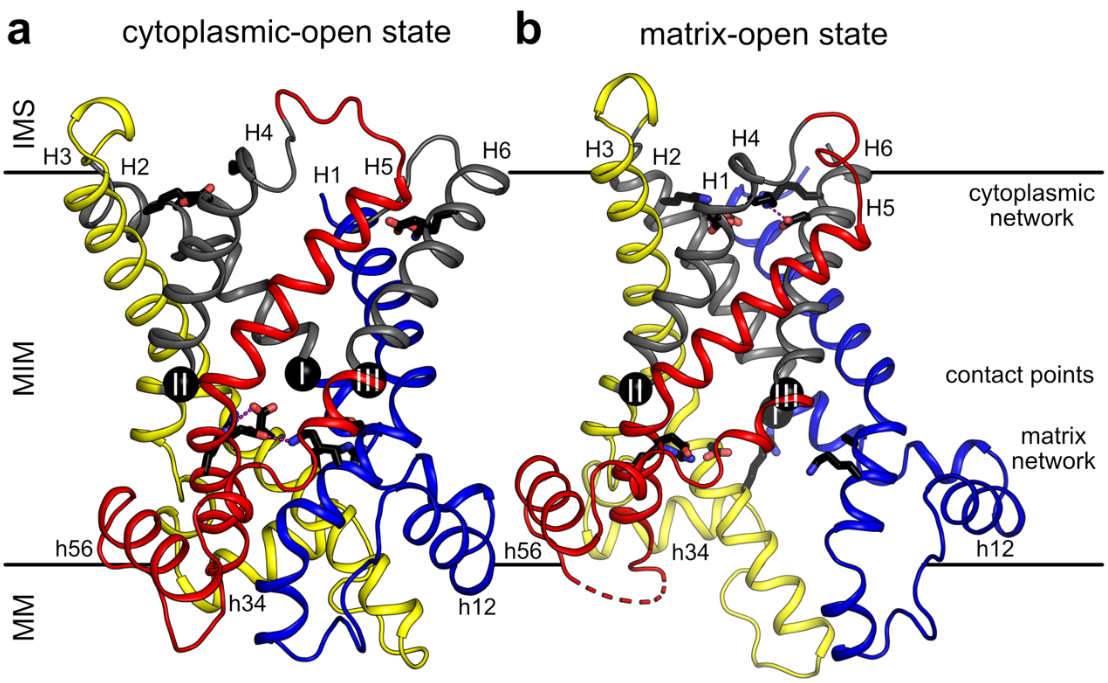

Supplementary Fig. 1 | Structures of the cytoplasmic and matrix state of the mitochondrial ADP/ATP carrier a, Membrane view of homology model of TtAac in the cytoplasmic-open state, generated with Modeller version 9.22, based on PDB codes 1OKC, 4C9H, 4C9Q, and 4C9J and **b**, the experimentally determined structure of TtAac in the matrix-open state (PDB code: 6GCI chain A). The core elements of domains 1, 2 and 3 are shown in blue, yellow and red, respectively, and the gate elements in grey. The three contact points of the substrate binding site[1,2] are shown as black spheres with roman numerals, which form also the hinge between the core and gate elements[3]. The cytoplasmic and matrix salt bridge networks are shown as black sticks with the ionic interactions of the formed network shown as magenta dashes.

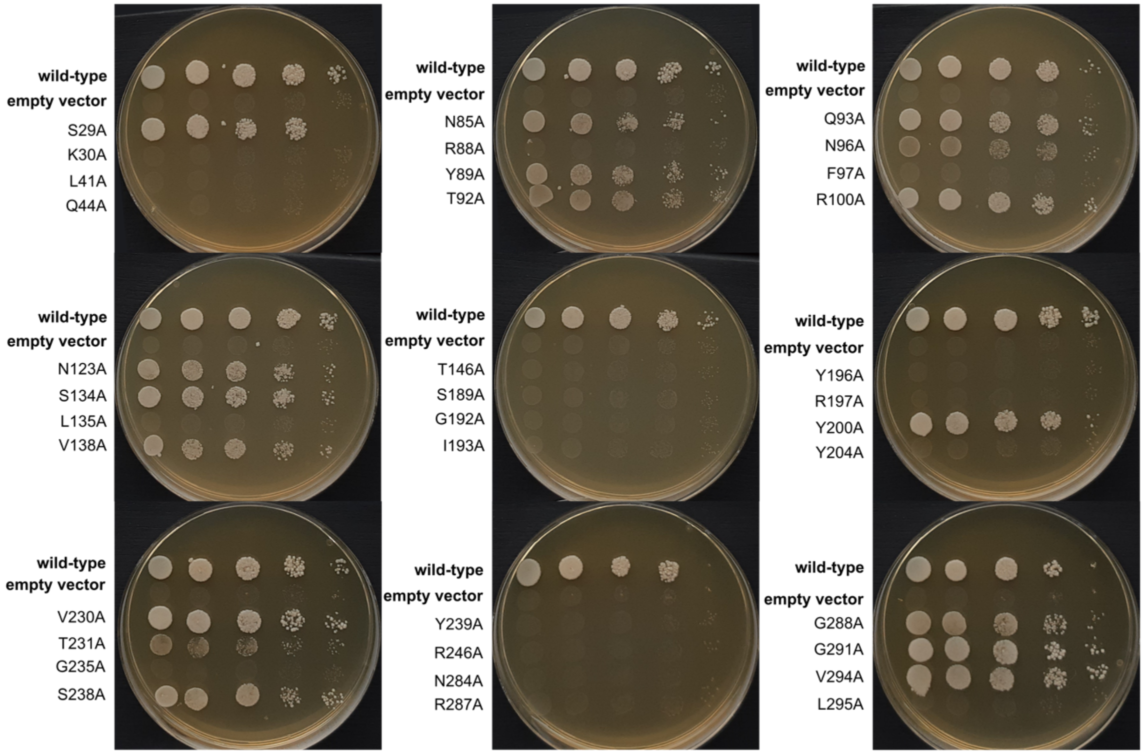

Supplementary Fig. 2 | Functional complementation assay of the WB-12 strain expressing wild-type or variant TtAac. The images depict one representative experiment (performed 4 times) for each variant. Wild type and empty vector controls are included in each plate as reference. From OD_{600}=1, four serial (1/10) dilutions were made and cells were grown on YPG medium, 30 °C for 72 h. The images are available at the Mendeley database under accession code doi:10.17632/mrhnw45w5y.1 and the generated densitometry data are provided in the Source Data file.

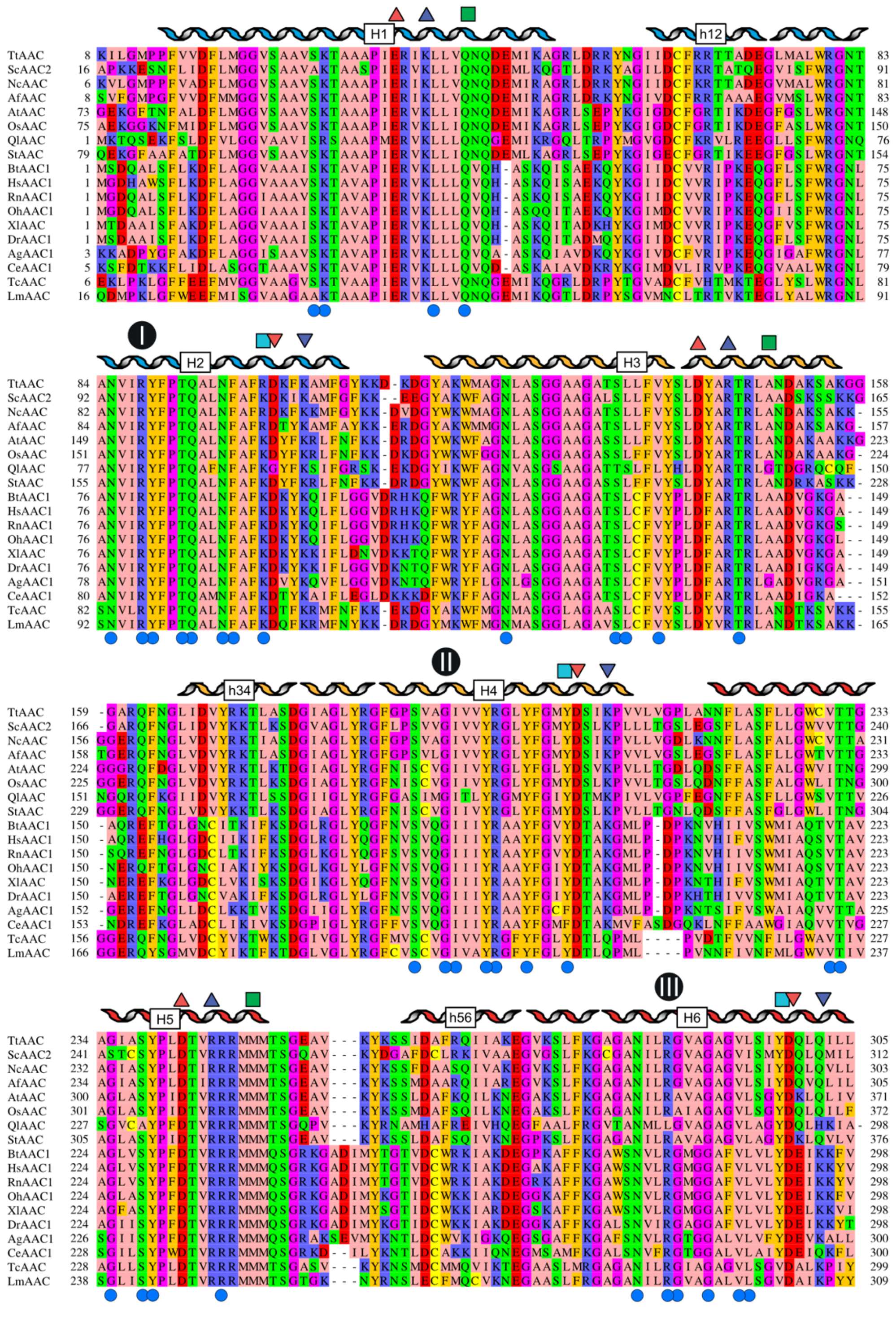

Supplementary Fig. 3 | Alignment of the amino acid sequences of selected mitochondrial ADP/ATP carriers. Alignment of the mitochondrial ADP/ATP carriers from the fungi *Thermothelomyces thermophila* (TtAac), *Saccharomyces cerevisiae* isoform 2 (ScAac2), *Neurospora crassa* (NcAAC), *Asparagillus fumigatus* (AfAAC), plants

Arabidopsis thaiana (AtAAC), *Oryza sativa* (OsAAC), *Querus lobata* (QlAAC), *Solanum tuberosum* (StAAC), animals *Bos Taurus* (BtAAC1), *Homo sapiens* (HsAAC1), *Rattus norvegicus* (RnAAC1), *Ophiophagus hannah* (OhAAC1), *Xenopus leavis* (XlAAC1), *Danio rerio* (DrAAC1), *Anopheles gambiae* (AgAAC1), *Caenorhabditis elegans* (CeAAC1), and the parasites *Trypanosoma cruzi* (TcAAC) and *Leishmania major* (LmAAC). Amino acids are colored according to their properties: basic K, R and H are blue, acidic D and E are red, polar N, Q, S and T are green, aliphatic A, I, L, M and V are pink, aromatic F, Y and W are orange, structural G and P are magenta, and C is yellow. The negatively charged (red) and positively charged (blue) residues of the matrix and cytoplasmic networks are indicated by up and down triangles, respectively. The positions of the glutamine brace (Q brace) and tyrosine brace (Y brace) are indicated by green and cyan squares. The contact points of the substrate binding site are shown in black circles with Roman numerals. The residues of the translocation pathway are indicated by blue spheres.

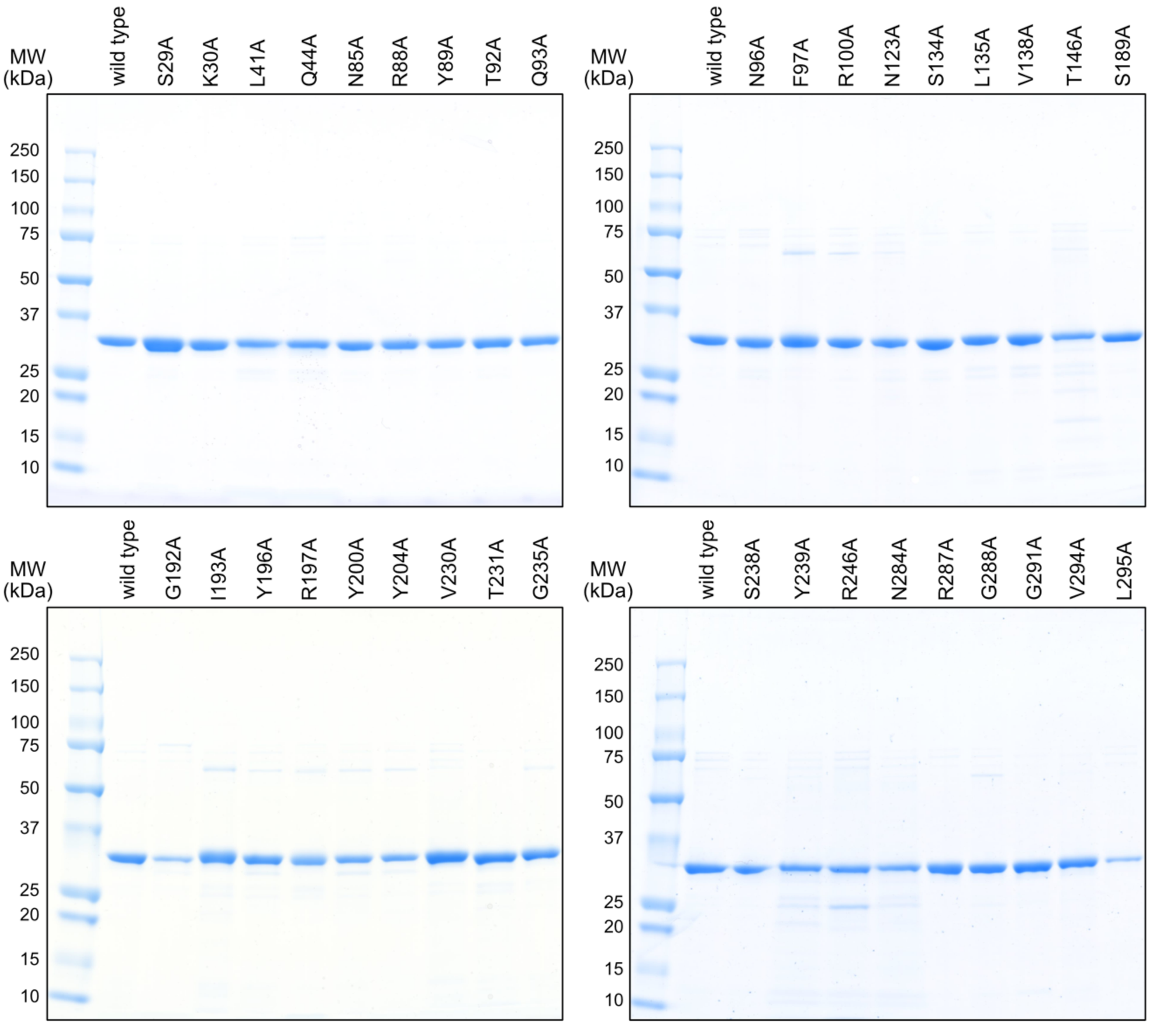

Supplementary Fig. 4 | Purification of TtAac wild-type and variants. Approximately 2 μg of each protein were analyzed by SDS–PAGE on 4-20% polyacrylamide gel and the bands were visualized by Coomassie Blue stain. Molecular weight (MW) markers are indicated. Gels were run after each independent purification, as reflected by the N numbers (Source Data file), and one representable sample for each protein was rerun here to show purity of the wildtype and variant proteins.

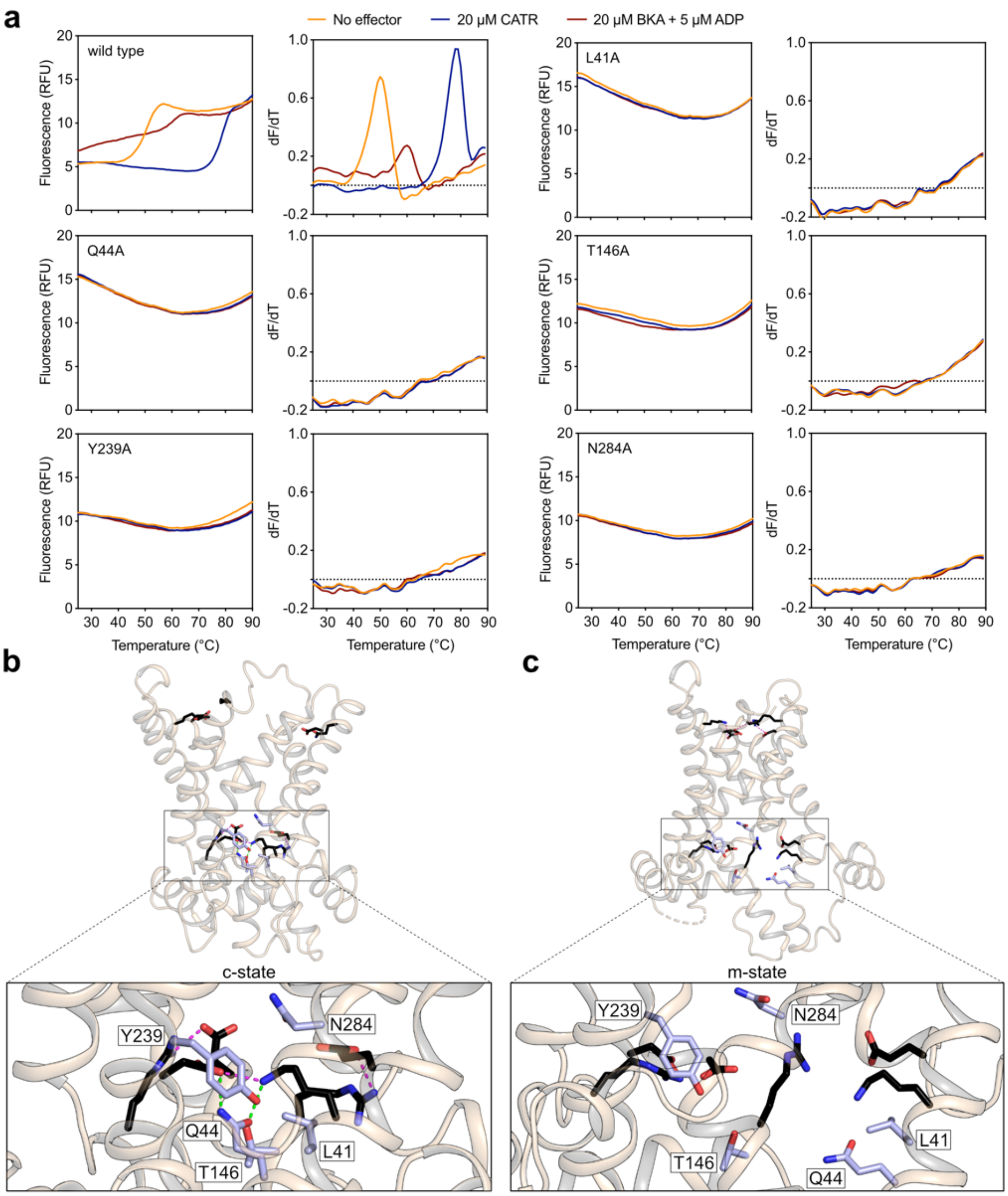

Supplementary Fig. 5 | Thermostability profiles and residue positions of the five unfolded variants. a, Typical thermal denaturation profiles of the wild type and the variants (left) and corresponding first derivative (right), indicating that the protein were unfolded after purification. The traces represent one representative experiment. Approximately 3 µg of protein were used for each condition. **b**, Membrane view of TtAac cytoplasm-open model (Fig. 1) and **c**, of experimentally determined matrix-open structure (PDB 6gci chain A). The residues which yield unfolded proteins after replacement with alanine are shown in violet sticks. Residues of the matrix and cytoplasmic networks are

shown as black sticks with ionic interactions shown as magenta dashes. The insets show details of the matrix gate environment in the two different states.

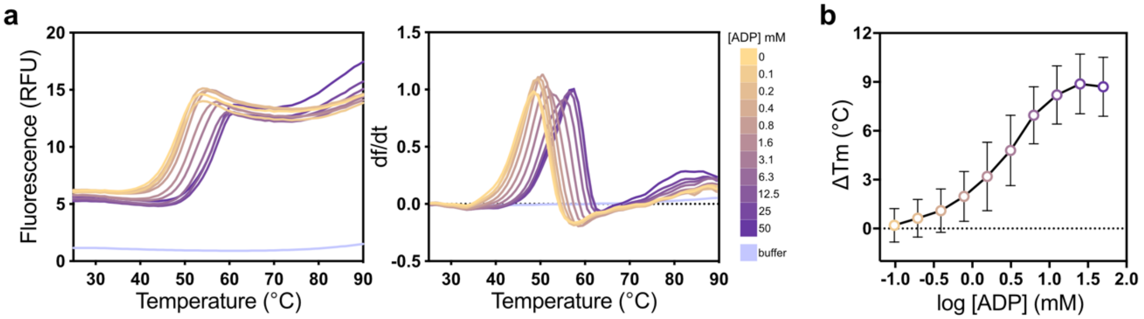

Supplementary Fig. 6 | Thermostability shifts in the presence of different concentrations of ADP for wild-type TtAac. a, Thermal denaturation profile (left) and corresponding first derivative (right) of the wild-type protein in presence of 0-50 mM ADP. Each unfolding trace is from one representative experiment. Approximately 3 μg of protein were used for each condition. **b**, Titration curve showing the thermostability shift (ΔTm) at each ADP concentration. The circles and error bars represent mean and standard deviation of 4 independent experiments.

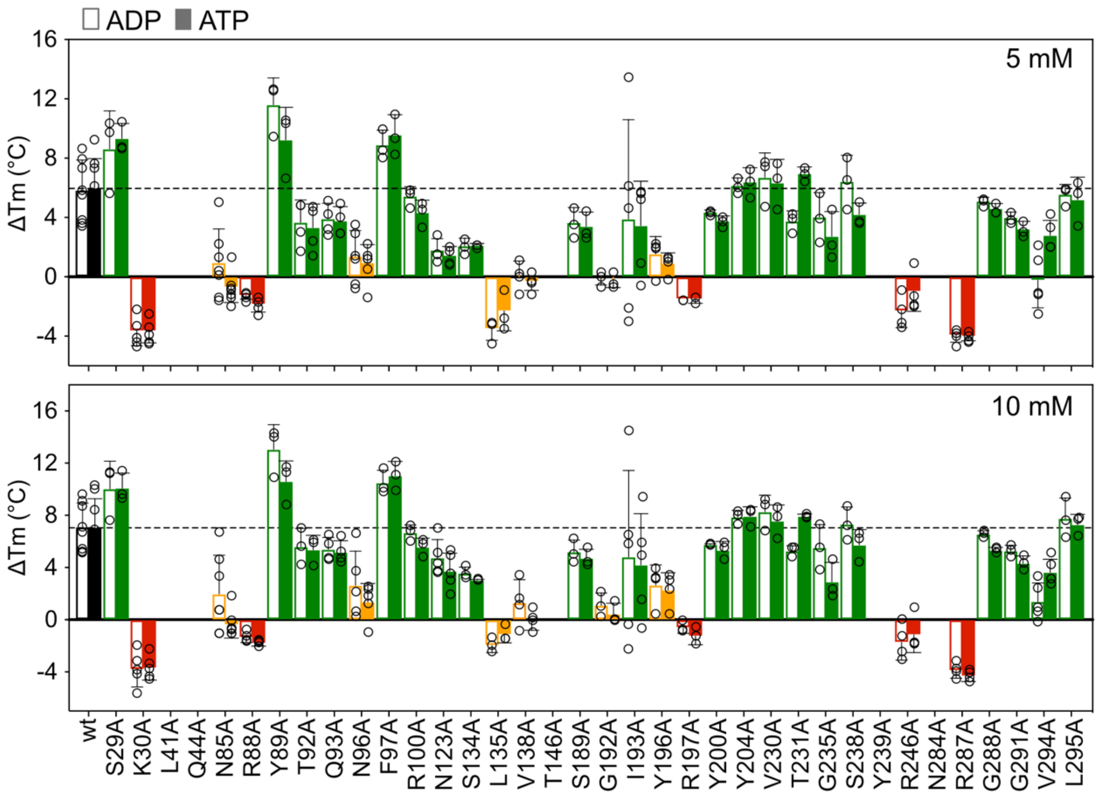

Supplementary Fig. 7 | ADP and ATP bind to the same set of residues in a similar way. Thermostability shift values (ΔTm) measured for 5 mM (top) and 10 mM (bottom) of ADP (empty bars) or ATP (filled bars). Bars and error bars represent the mean and standard deviation of at least three independent experiments, the N numbers and P values for the wildtype and variants are provided in the Source Data file. Empty and filled bars represent ADP and ATP, respectively. The colors of the bars are defined in Fig. 4 and 5 and relate to the response of the variants to substrate. Significance differences were evaluated by two-way ANOVA with interaction, as described in Materials and Methods. No significant differences in the shifts between ADP and ATP were observed for the wild type and variants at any concentration.

Supplementary References

1. Kunji, E.R. & Robinson, A.J. The conserved substrate binding site of mitochondrial carriers. *Biochim Biophys Acta* **1757**, 1237-48 (2006).
2. Robinson, A.J. & Kunji, E.R. Mitochondrial carriers in the cytoplasmic state have a common substrate binding site. *Proc Natl Acad Sci U S A* **103**, 2617-22 (2006).
3. Ruprecht, J.J. et al. The Molecular Mechanism of Transport by the Mitochondrial ADP/ATP Carrier. *Cell* **176**, 435-447.e15 (2019).
